# Supplementary material for: A Comprehensive Assessment of Ultraviolet-Radiation-Induced Mutations in Flammulina filiformis Using Whole-Genome Resequencing
Source: J Fungi (Basel). 2024 Mar 20;10(3):228. doi: 10.3390/jof10030228 (PMC10971301; doi:10.3390/jof10030228)
Supplement: Supplementary file 1 [file jof-10-00228-s001.zip › Supplementary Material S8/KEGG annotation/out/64381550635650.os/KO/out_map/map04962.html]

KEGG PATHWAY: Vasopressin-regulated water reabsorption - Reference pathway


|  |  |
| --- | --- |
| **Vasopressin-regulated water reabsorption - Reference pathway** |  |

[
Pathway menu
| Organism menu
| Pathway entry
| Show description
| User data mapping
]

In the kidney, the antidiuretic hormone vasopressin (AVP) is a critical regulator of water homeostasis by controlling the water movement from lumen to the interstitium for water reabsorption and adjusting the urinary water excretion. In normal physiology, AVP is secreted into the circulation by the posterior pituitary gland, in response to an increase in serum osmolality or a decrease in effective circulating volume. When reaching the kidney, AVP binds to V2 receptors on the basolateral surface of the collecting duct epithelium, triggering a G-protein-linked signaling cascade, which leads to water channel aquaporin-2 (AQP2) vesicle insertion into the apical plasma membrane. This results in higher water permeability in the collecting duct and, driven by an osmotic gradient, pro-urinary water then passes the membrane through AQP2 and leaves the cell on the basolateral side via AQP3 and AQP4 water channels, which are constitutively expressed on the basolateral side of these cells. When isotonicity is restored, reduced blood AVP levels results in AQP2 internalization, leaving the apical membrane watertight again.

|  |  |
| --- | --- |
| Reference pathway | 100% |
